# Peroral endoscopic mediastinal tunneling myotomy for esophageal achalasia: the first case treated in mediastinal tunnel

**DOI:** 10.1055/a-2308-2823

**Published:** 2024-05-17

**Authors:** Lijun Song, Liyun Ma, Li Wang, Guoliang Zhang, Ye Wang

**Affiliations:** 1 Department of Gastroenterology, Tianjin First Central Hospital, Tianjin, China; 2 Endoscopy Center and Endoscopy Research Institute, Zhongshan Hospital, Fudan University, Shanghai, China; 3 Department of Cardiology, Tianjin First Central Hospital, Tianjin, China

Peroral endoscopic myotomy (POEM) has been accepted as an effective and safe therapeutic strategy for esophageal achalasia 1 . However, the submucosal tunnel cannot be established in some patients because of severe fibrosis caused by previous treatment 2 .

A 68-year-old woman was admitted due to a 30-year history of progressive dysphagia and regurgitation. She was diagnosed with esophageal achalasia and had previously undergone two pneumatic balloon dilations, without significant improvement in symptoms. The esophagram revealed esophageal dilation and a “bird-beak” sign ( Fig. 1 ). Endoscopy revealed a massively dilated esophagus and narrow esophagogastric junction (EGJ) ( Fig. 2 ). POEM was planned.

**Fig. 1:**
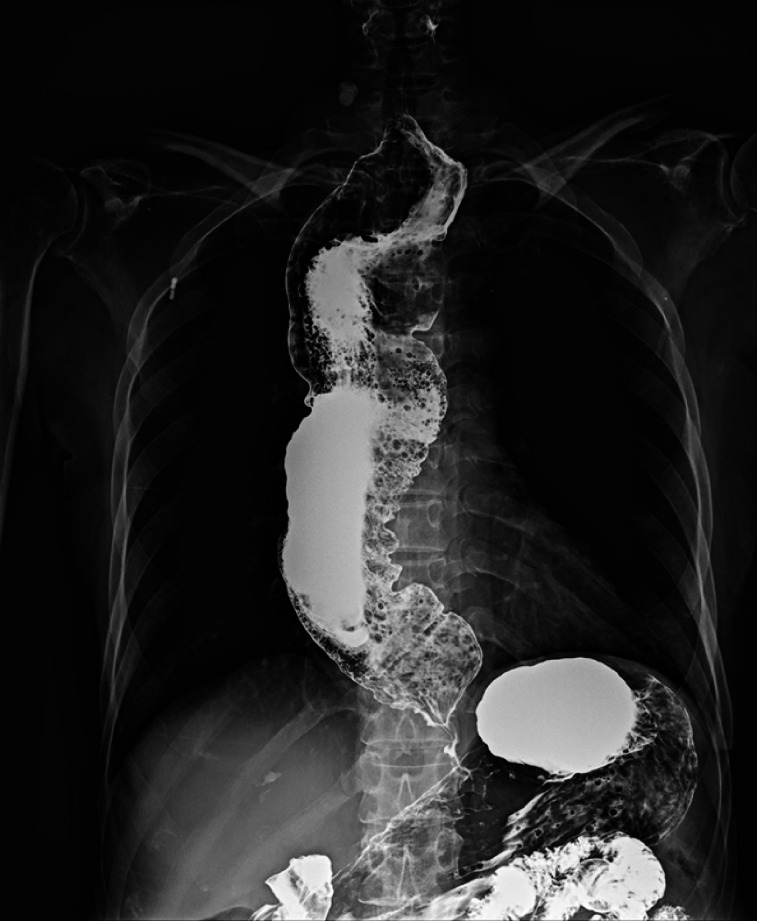
Barium esophagram before the procedure.

**Fig. 2:**
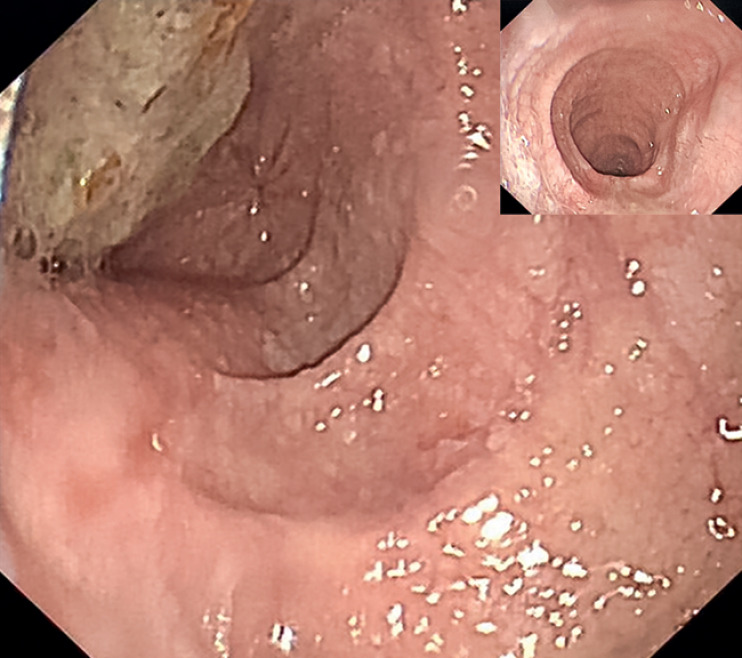
Endoscopy before the procedure.

Submucosal injections were administered 10 cm above the cardia, but resulted in no significant lift here or in other attempted injection sites. The submucosal adhesion in the esophagus was severe, making it impossible to establish a submucosal tunnel ( Fig. 3 ). A full-thickness incision of the esophageal wall was performed from the mucosal layer at 6 cm above the EGJ to create a mediastinal tunnel ( Video 1 ). Full-thickness myotomy started 5 cm above the EGJ and extended distally to 2 cm below the cardia in the mediastinal tunnel ( Fig. 4 ). The tunnel entrance was closed with six clips after hemostasis. A nasogastric tube was placed for decompression and monitoring.

**Fig. 3:**
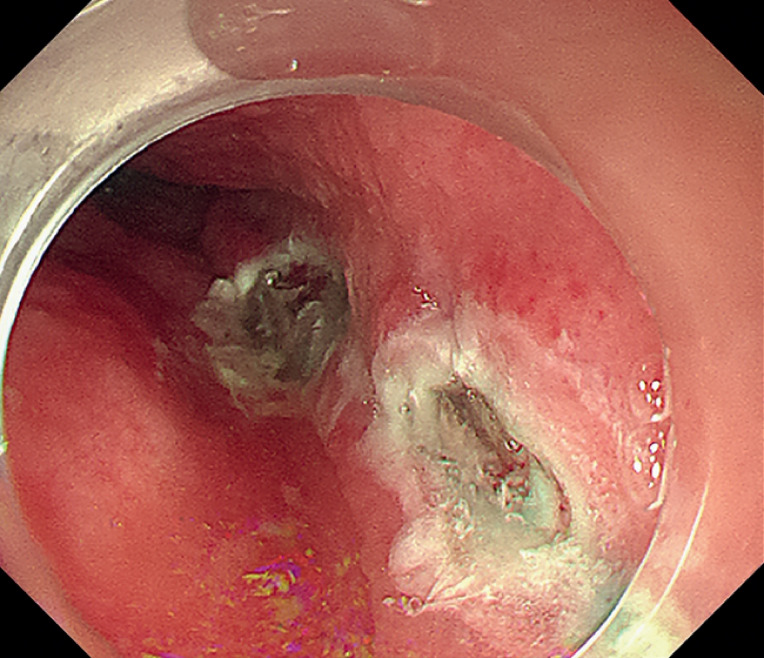
The submucosal tunnel could not be established due to severe submucosal fibrosis.

**Fig. 4:**
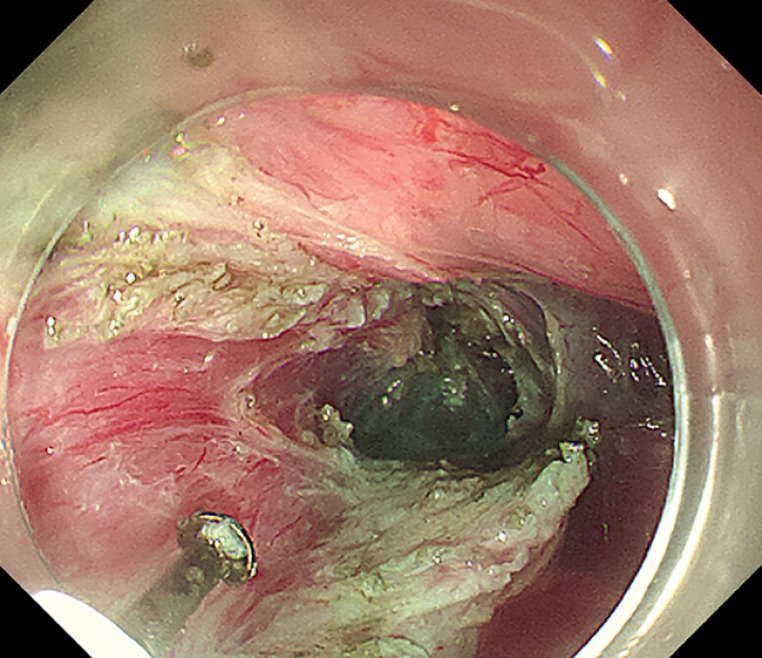
Full-thickness myotomy in the mediastinal tunnel.

Mediastinal dissection and tunneling.Video 1

The patient remained fasting and started a liquid diet after removing the nasogastric tube on postoperative day (POD) 3. She was able to take solid food on POD 5 and was discharged, uneventfully, on POD 7. A month later, endoscopy showed significant relaxation of the cardia ( Fig. 5 ).

**Fig. 5:**
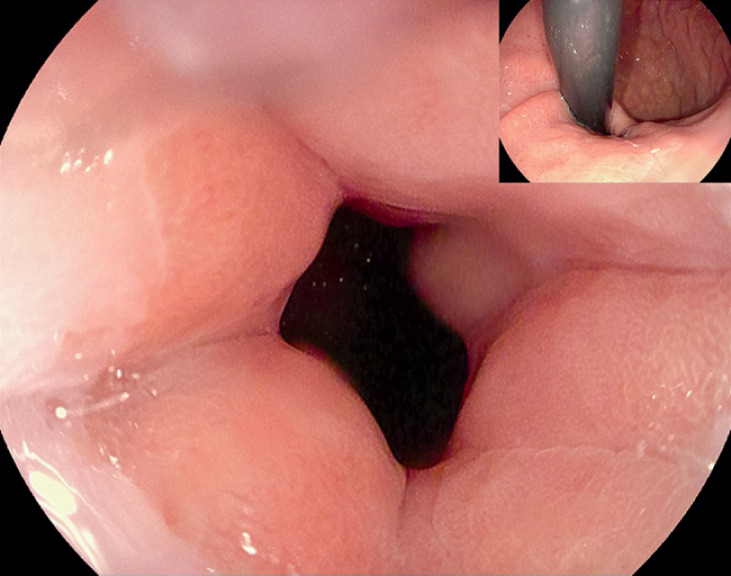
Endoscopy after the procedure.

Peroral endoscopic mediastinal tunneling myotomy (POEMTM) is a novel technique combining POEM and laparoscopic Heller myotomy, using the mediastinum for operating entry. In this video, we report the first application of POEMTM for achalasia with difficult submucosal tunnel, which may indicate a minimally invasive, safe, and effective alternative.

Endoscopy_UCTN_Code_TTT_1AO_2AG

## Notes

Endoscopy E-Videos https://eref.thieme.de/e-videos
*E-Videos* is an open access online section of the journal *Endoscopy* , reporting on interesting cases and new techniques in gastroenterological endoscopy. All papers include a high-quality video and are published with a Creative Commons CC-BY license. Endoscopy E-Videos qualify for HINARI discounts and waivers and eligibility is automatically checked during the submission process. We grant 100% waivers to articles whose corresponding authors are based in Group A countries and 50% waivers to those who are based in Group B countries as classified by Research4Life (see: https://www.research4life.org/access/eligibility/ ). This section has its own submission website at https://mc.manuscriptcentral.com/e-videos .
